# Designing bacterial signaling interactions with coevolutionary landscapes

**DOI:** 10.1371/journal.pone.0201734

**Published:** 2018-08-20

**Authors:** Ryan R. Cheng, Ellinor Haglund, Nicholas S. Tiee, Faruck Morcos, Herbert Levine, Joseph A. Adams, Patricia A. Jennings, José N. Onuchic

**Affiliations:** 1 Center for Theoretical Biological Physics, Rice University, Houston, Texas, United States of America; 2 Department of Chemistry & Biochemistry, The University of California, San Diego, California, United States of America; 3 Department of Biological Sciences, University of Texas at Dallas, Dallas, Texas, United States of America; 4 Department of Bioengineering, University of Texas at Dallas, Dallas, Texas, United States of America; 5 Department of Bioengineering, Rice University, Houston, Texas, United States of America; 6 Department of Biosciences, Rice University, Houston, Texas, United States of America; 7 Department of Physics & Astronomy, Rice University, Houston, Texas, United States of America; 8 Department of Pharmacology, The University of California, San Diego, California, United States of America; 9 Department of Chemistry, Rice University, Houston, Texas, United States of America; Weizmann Institute of Science, ISRAEL

## Abstract

Selecting amino acids to design novel protein-protein interactions that facilitate catalysis is a daunting challenge. We propose that a computational coevolutionary landscape based on sequence analysis alone offers a major advantage over expensive, time-consuming brute-force approaches currently employed. Our coevolutionary landscape allows prediction of single amino acid substitutions that produce functional interactions between non-cognate, interspecies signaling partners. In addition, it can also predict mutations that maintain segregation of signaling pathways across species. Specifically, predictions of phosphotransfer activity between the *Escherichia coli* histidine kinase EnvZ to the non-cognate receiver Spo0F from *Bacillus subtilis* were compiled. Twelve mutations designed to enhance, suppress, or have a neutral effect on kinase phosphotransfer activity to a non-cognate partner were selected. We experimentally tested the ability of the kinase to relay phosphate to the respective designed Spo0F receiver proteins against the theoretical predictions. Our key finding is that the coevolutionary landscape theory, with limited structural data, can significantly reduce the search-space for successful prediction of single amino acid substitutions that modulate phosphotransfer between the two-component His-Asp relay partners in a predicted fashion. This combined approach offers significant improvements over large-scale mutations studies currently used for protein engineering and design.

## Introduction

Designing mutations that encode new interactions between non-partner proteins is a powerful strategy for developing new approaches to problems in diverse areas in systems biology. For example, rewiring signaling pathways to get a desired response or restore healthy response to a route that was damaged by disease is a grand-challenge in global health. Large-scale mutational-selection studies are currently employed but are only amenable to a few select systems. What is needed is a more directed data-driven strategy. However, a large bottleneck in these design efforts is the relative paucity of structural and dynamic information on proteins and protein variants that mediate interactions, relative to the wealth of genomic data available, which is increasing at exponential rates. Towards the goal of streamlining design efforts for new protein partners, we focus on the ubiquitous bacterial signal transduction systems, two-component signaling (TCS) systems, and the wealth of available sequence data.

The guided redesign of TCS components to either activate or inhibit cross species signaling in a is a grand challenge in both protein design and synthetic biology. The successful achievement of guided design is an essential as a first step towards engineering new, desired properties into diverse systems of interest. However, the traditional approach of examining the surfaces that modulate protein-protein interactions and catalysis is not a robust path forward. While alternatives such as shotgun mutagenesis and selection or scanning mutagenesis can lead to rewiring *in vitro* [1, 2] and *in vivo* [2], it is clear that a brute force strategy for selecting mutations [3] is not uniformly practical. Moving forward, it is essential that the design of new non-cognate protein-protein interactions include economical, data-driven approaches. Towards this goal, we model abundant sequence data using Direct Coupling Analysis (DCA) as a guide to design interactions between enzymes and their targets that are non-cognate partners.

**Fig 1:**
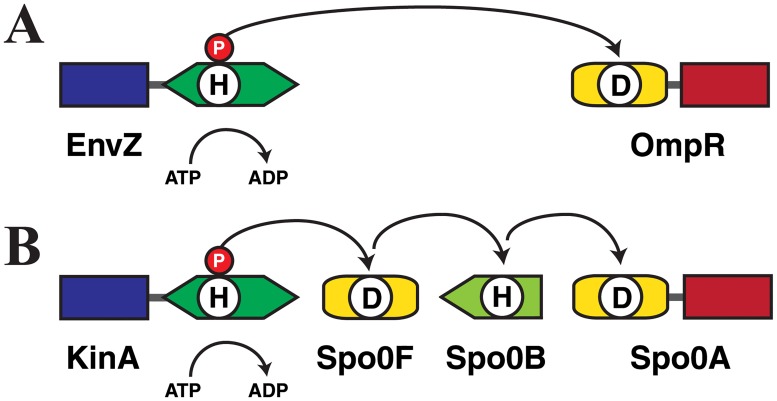
Protein Domain Architecture of Stress-Response His-Asp Phosphorelay Systems. (A) *Escherichia coli* osmoregulatory system. The sensor HK EnvZ transfers a phosphoryl group to the transcription-factor-type RR OmpR. (B) *Bacillus subtilis* sporulation control system. Phosphotransfer occurs from the sensor HK KinA, via the RR Spo0F and the histidine phosphotransfer protein Spo0B, to the transcription-factor-type response regulator Spo0A.

In the current work, we chose the bacterial histidine kinase (HK) EnvZ from *Escherichia coli* and the response regulator (RR) Spo0F from *Bacillus subtilis* as our system of choice (Fig 1). Bacteria live in constantly changing environments, as they must continuously monitor external conditions in order to adjust their shape, motility and physiology to survive and thrive. The histidine-aspartate phosphorelay TCS systems [4–9] are important sentries in monitoring the cellular environment and guiding cellular adaptation to environmental changes. Thus, the HK protein function as both a sensor and signal transducer. The HK EnvZ responds to osmotic stress by first autophosphorylating then quickly relaying the phosphoryl group to the transcription-factor like RR protein OmpR, to elicit an environmental-stress response (Fig 1A). There are as many as 10^2^−10^3^ homologous TCS systems in bacteria, with each controlling the response to a different stimulus. For example, in a related, but multistep relay reaction, the HK KinA shuttles a phosphate through the RR Spo0F to the final RR Spo0A, to initiate sporulation in *Bacillus subtilis* (Fig 1B). HK proteins interact preferentially with their own partner RRs, where the ability to bind preferentially to one another is encoded by the complementary interaction surface residues, although non-partner signal transfer (“cross-talk”) has sometimes been observed *in vitro* [10, 11]. Therefore, the HK-RR protein partner systems are ideal candidates to explore the challenge of using sequence data alone to tune the activation or inhibition of cross species reactivity.

Earlier work [1, 2, 12–17] has successfully used statistical approaches to find evolutionarily conserved interactions between HK and RR partners from information encoded in the multiple sequence alignments (MSA) of cognate TCS partners. These studies were able to quantify the correlated amino acid identities between the HK and RR partner proteins that arise from the constraint maintain their functional interactions (i.e., amino acid coevolution), which can be used to identify those functional residue interactions. In particular, DCA-based approaches have been successful in describing the specificity of interaction between HK and RR cognate partner proteins [13, 15, 16, 18, 19], and successfully provided spatial constraints for prediction of the 3D TCS structure [12]. In addition, related approaches were validated for diverse protein structure prediction problems [20–24]. However, identifying regions and residue identities amenable to rewiring of non-cognate interactions has remained a significant challenge. Towards this goal, we use data-driven Direct Coupling Analysis (DCA) as a method of choice to design interactions between enzymes that are non-cognate partners. We test our predictions with controlled biochemical methods rather than brute-force selection strategies as a means to reduce the workload needed to identify promising candidate partners. We selected wild-type EnvZ from *Escherichia coli*, as the phosphodonor and a series of mutant RR Spo0F proteins from *Bacillus Subtilis* predicted to have a range of phosphoacceptor efficiencies with the non-cognate partner from a distinct organism. Our experimental results validate that a co-evolutionary approach, purely based on sequence, for selecting mutations with specific predicted changes in activity offers significant improvements over large-scale mutations studies currently used for protein engineering and design.

## Results

### Inferring candidate mutations for the RR Spo0F

Candidate mutations of the RR Spo0F predicted to encode preferential interaction with the HK EnvZ were selected from a subset of mutations for which $\Delta H_{\text{TCS}}=H_{\text{TCS}}({\vec{\sigma }}_{\text{mutant}})-H_{TCS}({\vec{\sigma }}_{w.t.})<0$ (Eq 1), where ${\vec{\sigma }}_{\text{mutant}}$ and ${\vec{\sigma }}_{\text{w}.\text{t}.}$ are the mutated and wild-type sequences, respectively. These mutations are interpreted as increasing the signal transfer efficiency between EnvZ and Spo0F, according to the inferred quantitative model [18].

**Fig 2:**
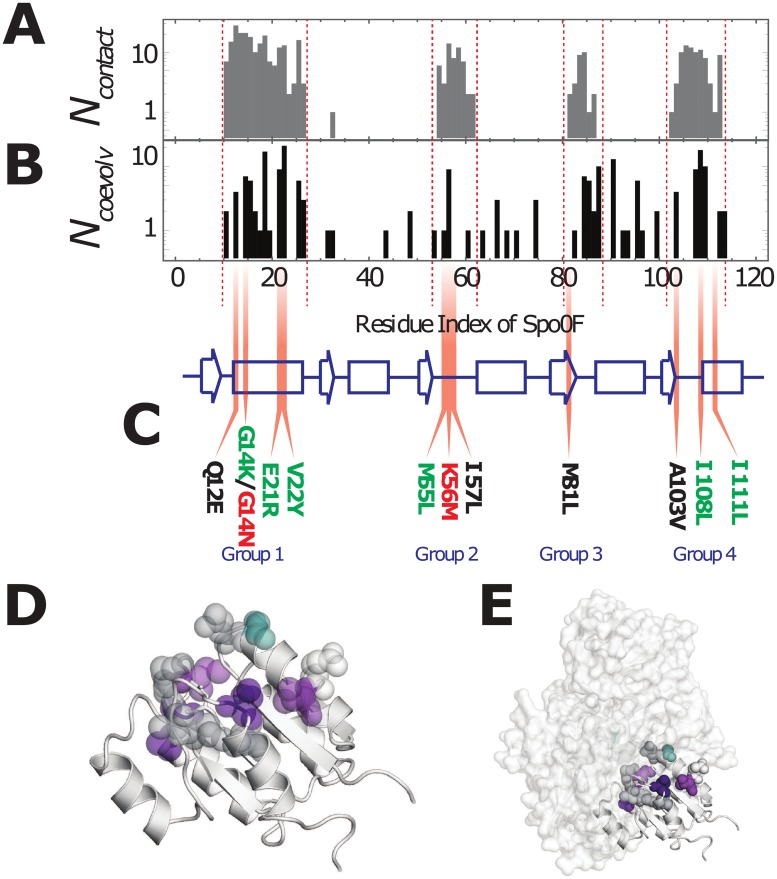
Selecting mutations from highly coevolved regions of the TCS interface. (A) A histogram of the number of contacts, *N*_contact_, formed between the RR Spo0F and the HK KinA in a representative structure of the TCS complex is plotted as a function residue number on Spo0F [16]. (B) Using coevolutionary analysis of HK/RR partner sequences, the top 200 coevolving HK/RR interprotein residue pairs are calculated using Direct Information (DI) (Eq 2). For these top coevolving residue pairs, the number of HK residues coevolving with each RR residue, *N*_coevolv_, is plotted in a histogram as a function of the residue numbers of the RR protein family, which are mapped on to the corresponding residue numbers on Spo0F. (C) The secondary structure of Spo0F is drawn as a cartoon, with strands denoted by arrows, helices by rectangles and loops and turns denoted by lines. Mutations in highly coevolving RR residues that formed contacts with the HK in this study were obtained from the four groups and are shown in Gray (Group 1), Red (Group 2), Cyan (Group 3), and Blue (Group 4). (D) The mutations (colored by group) are plotted on the representative structure of Spo0F (PDB ID: 1PEY) [37]. (E) The mutations (colored by group and represented as spheres) are shown on Spo0F bound to KinA in the representative TCS complex [16]. The structural representations in (D) and (E) were generated using PyMOL [45].

Selected mutations are limited to single residue sites on the RR Spo0F that are predicted to (*i*) form contacts with its partner [25] HK KinA from *Bacillus subtilis* in a representative TCS complex and (*ii*) coevolve with the residues of the HK. A representative TCS complex formed by the HK KinA and Spo0F was predicted using the coevolutionary couplings observed in multiple sequence alignments of the HK/RR partners [16]. This data is consistent with experimental structures [26]. Fig 2A shows the number of contacts, *N*_contact_, formed between Spo0F and KinA in the representative structure using a 10Å cutoff between all heavy atoms. Four main groups of residues on Spo0F form the contacts with the HK KinA, i.e., α1 (Group 1), β3-α3 loop (Group 2), β4/β4- α4 loop (Group 3), and β5- α5 loop/α5 (Group 4).

The correlated amino acid identities between inter-protein residue pairs (i.e., HK/RR residue pairs) observed in the sequence data are quantified using the Direct Information (DI) (Eq 2) [13, 20], which can be ranked from highest (highly coevolving) to lowest (uncorrelated). Highly coevolving interprotein residue pairs between the HK and RR form contacts that stabilize the TCS complex [12, 13, 16]. Fig 2B shows the number of HK residues found to strongly coevolve with each RR residue, *N*_coevolv_. Spo0F residue positions with a high *N*_coevolv_ are interpreted as being candidate sites for encoding new TCS interactions.

The primary candidates for enhancing the phosphotransfer between EnvZ and Spo0F were chosen from the overlap between Fig 2A and 2B, for mutations that satisfy Δ*H*_TCS_ < 0 (Eq 1). These primary candidates are G14K, E21R, and V22Y from Group 1, M55L from Group 2, and I108L and I111L from Group 4. While 5 of 6 of these mutations match the wild-type residues of the RR OmpR, the cognate partner of the EnvZ kinase, simply copying the amino acid identities of OmpR is insufficient (S1 Text). Additional mutations are also selected from the four contact groups. The mutations G14N and K56M were chosen because they are predicted to be highly deleterious to phosphotransfer between EnvZ and Spo0F, i.e., Δ*H*_TCS_ > 0. The remaining mutations that were explored include Q12E, I57L, M81L, and A103V, which are predicted to have a neutral effect on the phosphotransfer between EnvZ and Spo0F. Fig 2C shows all of the selected mutations on the secondary structural elements of Spo0F. Fig 2D shows all of the single-site mutations plotted together on the representative structure of Spo0F. All of these mutational sites physically interact with the HK in the representative structure of a TCS complex (Fig 2E).

**Fig 3:**
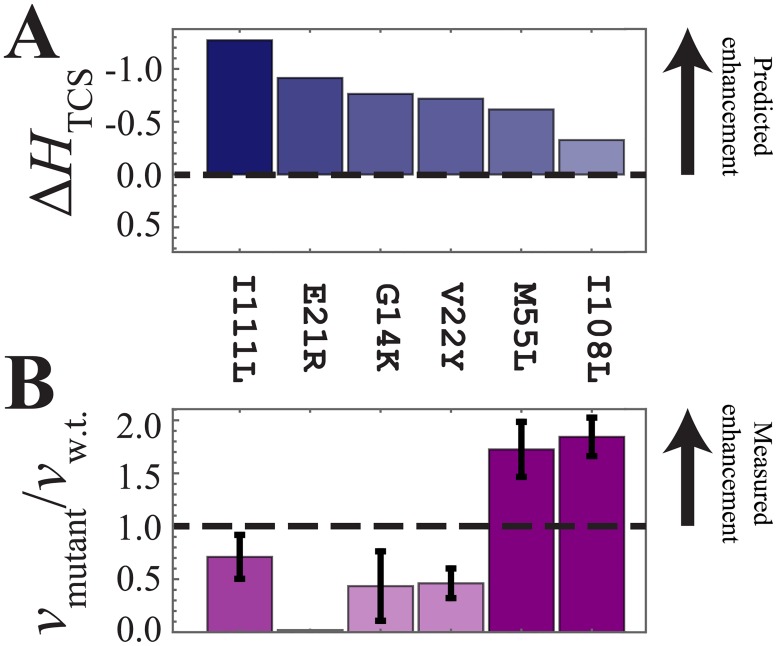
Experimental analysis of the mutants predicted to enhance phosphotransfer. (A) The computational model predicts that I108L, M55L, I111L, V22Y, G14K, and E21R will enhance phosphotransfer between HK EnvZ and RR Spo0F, i.e., Δ*H*_TCS_ < 0. (B) The *in vitro* phosphotransfer rate between EnvZ and each of the Spo0F mutations, *v*_mutant_, is plotted normalized by the phosphotransfer rate between wild-type EnvZ/Spo0F, *v*_w.t._. Here, *v*_mutant_ / *v*_w.t._ > 1 shows mutations that enhanced the phosphotransfer rate. The mutations I111L, E21R, and V22Y are found to be destabilizing with respect to the wild-type Spo0F stability (Table 1), therefore it is not a surprised that they cannot increase the phosphotransfer rate. M55L and I108L did not result in significant destabilization of Spo0F and were found to successfully enhance phosphotransfer in agreement with our theoretical predictions.

**Fig 4:**
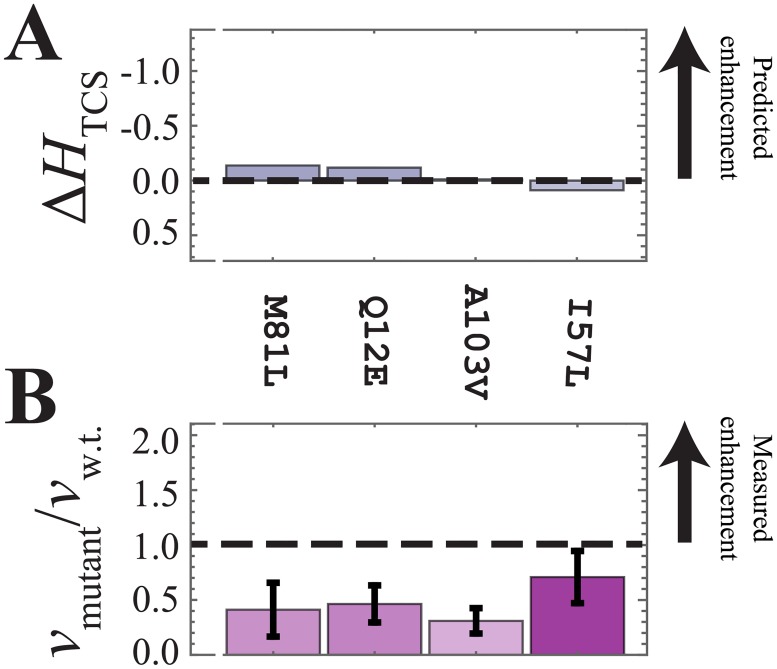
Experimental analysis of the mutants predicted to have a neutral effect on phosphotransfer. (A) The computational model predicts that I57L, Q12E, M81L, and A103V will have a neutral effect on the phosphotransfer, i.e., Δ*H*_TCS_ ≈ 0. (B) The four mutants were observed to result in a decrease in phosphotransfer compared to the wild-type EnvZ/Spo0F interaction. Of these four mutations, only A103V was not found to significantly destabilize Spo0F (Table 1).

**Fig 5:**
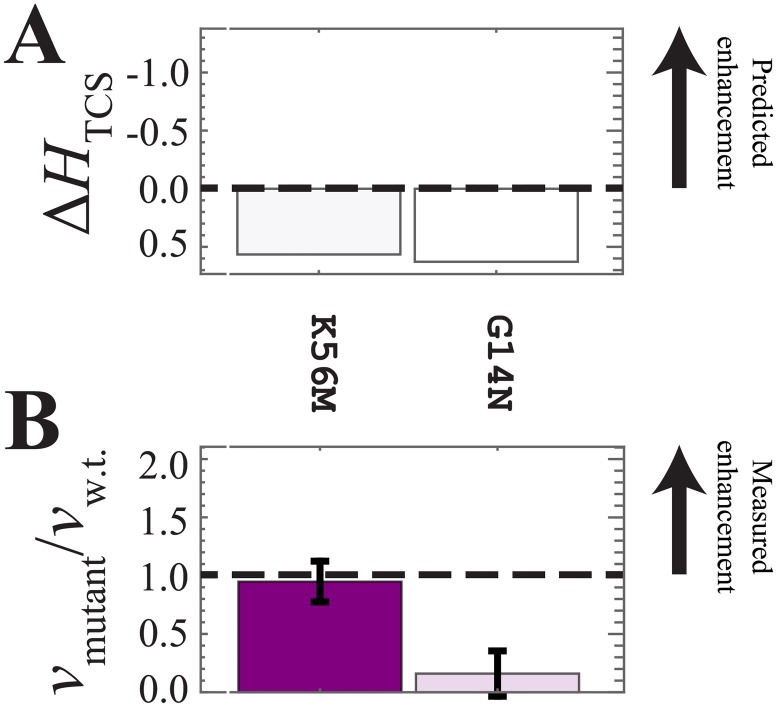
Experimental analysis of the mutants predicted to suppress phosphotransfer. (A) The computational model predicts that K56M and G14N will suppress phosphotransfer, i.e., Δ*H*_TCS_ > 0. (B) K56M exhibited comparable phosphotransfer to the wild type, while G14N showed significant suppression of phosphotransfer.

The computational predictions of the signal transfer efficiency, Δ*H*_TCS_, are shown in Figs 3A, 4A and 5A. Finally, Figs 3B, 4B and 5B shows the experimental phosphotransfer results, which are discussed in the following subsection (*vide infra*).

### Experimental analysis of the mutants predicted to enhance phosphotransfer

Fig 3A shows the predicted signal transfer efficiency, Δ*H*_TCS_, for the mutations G14K, E21R, V22Y, M55L, I108L and I111L, which are predicted to enhance phosphotransfer with respect to the wild-type EnvZ/Spo0F interaction. The experimental phosphotransfer reaction between EnvZ/Spo0F (wild-type and mutated proteins) is carried out *in vitro* to obtain phosphotransfer rates for the wild-type and mutated Spo0Fs, denoted as *v*_w.t._ and *v*_mutant_, respectively. The ratio of the relative phosphotransfer rates of the mutated to the wild-type phosphotransfer rate, *v*_mutant_ / *v*_w.t._, are shown in Fig 3B. Of the 6 candidate mutated proteins, M55L and I108L are experimentally confirmed as true positive predictions (i.e., *v*_mutant_ / *v*_w.t._ > 1), I111L maintained wild-type activity, while V22Y, G14K and E21R did not enhance phosphotransfer efficiency. Given that the E21R protein exhibits significantly decreased stability, Table 1, offers a plausible explanation as to why this HK/RR pair was instead found to decrease the *in vitro* phosphotransfer activity.

**Table 1:** The enthalpy (Δ*H*_DSC_) of unfolding measured using Differential Scanning Calorimetry (DSC).

Spo0F mutational variant	Enthalpy of unfolding, Δ*H*_DSC_ (10^4^ kJ/mol)
Wild-type	1.80 ±0.19
G14K	2.02 ±0.36
E21R	0.01 ±0.04
V22Y	1.49 ±0.15
M55L	2.01 ±0.18
I108L	2.48 ±0.04
I111L	0.62 ±0.13
Q12E	1.08 ±0.04
I57L	0.96 ±0.20
M81L	0.84 ±0.05
A103V	2.53 ±0.04
G14N	1.56 ±0.04
K56M	1.25 ±0.05

The table is divided into four groups, wild-type, and enhanced activity, neutral effect on activity, and suppressed activity (top to bottom, respectively).

### Experimental analysis of the mutants predicted to have a neutral effect on phosphotransfer efficiency

Fig 4A shows the DCA predictions for the mutations Q12E, I57L, M81L, and A103V, which are expected to have a neutral effect on the phosphotransfer reaction. The ratio of the relative mutant phosphotransfer rates of the mutated to the wild-type phosphotransfer rate, *v*_mutant_ / *v*_w.t._, are shown in Fig 4B, revealing that the mutants Q12E, M81L, and A103V all exhibited significant decrease in the phosphotransfer rate. Only I57L exhibited comparable phosphotransfer to that of the wild-type Spo0F. The observed change in the enthalpy of unfolding, Δ*H*_DCS_, for Q12E, I57L, and M81L indicates significant destabilization of the proteins relative to the WT (Table 1), offering a plausible explanation as to why only the I57L HK/RR pair had a neutral effect on phosphotransfer efficiency.

### Experimental analysis of the mutants predicted to suppress phosphotransfer efficiency

Fig 5A shows the DCA predictions of signal transfer efficiency for the mutations G14N and K56M, which are predicted to suppress phosphotransfer. The ratio of the relative mutation phosphotransfer rates of the mutant to the wild-type phosphotransfer rate, *v*_mutant_ / *v*_w.t._, are shown in Fig 5B. While G14N is correctly predicted to result in a significant decrease in phosphotransfer efficiency, K56M is found to exhibit comparable phosphotransfer to the wild-type. These DCA predictions rely on the need of sufficient sequences that co-vary at the site of the specific RR mutation with residues of the HK in order to provide good predictions. This is always the case when we observe a negative Δ*H*_TCS_ and therefore the method is powerful in predicting mutants that increase phosphotransfer efficiency. That is, a positive Δ*H*_TCS_ may be a consequence of bad statistics and can only correctly predict a decrease of phosphotransfer efficiency when sufficient sequences are available.

## Discussion

Guided protein design is an essential tool as a first step towards engineering new, desired properties into TCS systems. Ideal candidates for enhancing activity can be selected from the subset of mutations that both are predicted to enhance phosphotransfer, while not significantly destabilizing the RR Spo0F such that they aggregate with respect to the wild-type protein. The current work selects mutations directly from a coevolutionary landscape of TCS partners, Δ*H*_TCS_, which serves as a proxy for signal transfer efficiency between a specific HK and RR [18], even if they are not cognate partners.

The inferred model, Δ*H*_TCS_, is a fitness landscape that describes amino acid selection observed in sequences of TCS partners. The pairwise couplings of the landscape capture the amino acid coevolution between residues of the HK and RR to maintain functional signaling between partner proteins in bacteria. These couplings not only capture the amino acid identities that lead to preferential binding, but also the inter-protein residue interactions involved in the chemistry of efficiently transferring a phosphoryl group. We focus on mutating the variable residue sites of the RR that are highly coevolving with residues of the HK, which are involved in binding and recognition; on the other hand, residues involved in the phosphotransfer reaction tend to be conserved [16]. Related works have also extended the analysis of coevolutionary information to explore the phenotype-genotype relationship, mutational epistasis, and fitness landscapes [27–29] as well as the identification of interactions in a protein-interaction network [30].

While this model was able to predict multi-point mutations within a TCS protein that could maintain its functionality [18], in agreement with the mutations found in experiment [31], it was not known if it could be used to select new partners by design, in the absence of additional structural data. In this work, we turn our attention towards using the model to design new TCS interactions. Mutations are selected to both enhance and maintain the signal transfer between the HK EnvZ from *E*. *coli* and the RR Spo0F from *B*. *subtilis*. These results show that 2 of the 6 mutations predicted to enhance EnvZ/Spo0F signal transfer in silico succeed to do so *in vitro*. Taking into consideration that mutations that significantly destabilize the native protein fold enhances the predictive power of our approach. Of the mutations predicted to enhance phosphotransfer activity while maintaining stability, 2 out of the 5 mutations succeed as E21R is significantly destabilized relative to the WT protein and has a propensity to aggregate (Table 1).

While it is possible to generate and interrogate the functionality of hundreds of thousands of mutations through traditional high-throughput methods *in vivo* [31], these approaches are time consuming and wasteful. Further, simple comparison of multiple sequence alignments over related species alone to generate testable mutations excludes the entire sequence space sampled *in vivo* for the TCS proteins. Our design of new TCS interactions is significantly enhanced using data-driven, computational approaches. Due to the low computational cost of generating predictions using Δ*H*_TCS_, one readily can search sequence-space for amino acid combinations that enhance signal transfer between non-cognate partners. This combined computational and experimental approach complements existing strategies for engineering bacterial responses that are based on modular design [32–36].

## Materials and methods

### Model structure of EnvZ/Spo0F complex

The detailed crystal structure of a TCS complex was first obtained for HK853/RR468 of *Thermatoga maritima* [26], elucidating the binding interface between HK and RR partners. It was subsequently shown [12] that TCS complexes could be computationally predicted using highly coevolving interprotein (HK/RR) residue pairs as docking constraints for molecular dynamics simulations. In this present work, the computationally predicted structure for the KinA/Spo0F complex (*B*. *subtilis*) [16] is used as a model for selecting mutations to stabilize the EnvZ/Spo0F complex. This predicted complex is composed of a representative structure for the Spo0F monomer, obtained from crystallography (PDB ID: 1PEY) [37], and a representative structure for the KinA homodimer, obtained from homology modeling using I-TASSER [38]. The sequence of EnvZ is threaded into the template structure of KinA.

### Direct Coupling Analysis (DCA)

Multiple-sequence alignments (MSA) of the HK (PF00512) and RR (PF00072) protein families are collected from Pfam [39] (Version 28). The HK and RR aligned sequences are then concatenated based on genomic adjacency [10, 40]. The concatenated sequence of amino acids for a TCS partner pair, $\vec{\sigma }=(\sigma_{1},\sigma_{2},\ldots ,\sigma_{L})$, is represented as a vector of length *L* = 172 where amino acids 1 to 60 and 61 to 172 belong to the HK and RR, respectively. Additional details of the TCS partners used to parameterize the coevolutionary model can be found in Ref. [18].

Methods such as Direct Coupling Analysis (DCA) [13, 20, 21] infer a probabilistic model, $P(\vec{\sigma })=\text{exp}\left(-H(\vec{\sigma })\right)/Z$, for the selection of the sequence data, $\vec{\sigma }=(\sigma_{1},\sigma_{2},\ldots ,\sigma_{L})$. The approach adopted in this study uses pseudolikelihood maximization [21] to infer the statistical couplings, *j*_*ij*_, and local fields, *h*_*i*_, of a Potts model, $H(\vec{\sigma })=-\sum_{i<j}J_{ij}(\sigma_{i},\sigma_{j})-\sum_{i}h_{i}(\sigma_{i})$.

### Construction of the TCS coevolutionary landscape

Focusing on the interprotein couplings between the HK and RR residues, a proxy for signal transfer efficiency between TCS proteins was constructed:

$$H_{\text{TCS}}(\vec{\sigma })=-\sum_{i=1}^{60}\sum_{j=61}^{172}J_{ij}(\sigma_{i},\sigma_{j})\times \Theta (c-r_{ij})-\sum_{i=1}^{172}h_{i}(\sigma_{i})$$ (1)

where $\vec{\sigma }$ is the concatenated sequence of wild-type EnvZ (HK) and wild-type or mutated Spo0F (RR), the double summation is taken between all interprotein residue pairs (i.e., residues 1 to 60 and 61 to 172 belonging to the HK and RR, respectively), Θ is a Heaviside step function, *c* is a cutoff distance of 16Å, and *r*_*ij*_ is the minimum distance between residues *i* and *j* in the representative structure. Mutational changes in Eq 1 are expressed as $\Delta H_{\text{TCS}}(\vec{\sigma })=H_{\text{TCS}}({\vec{\sigma }}_{\text{mutant}})-H_{\text{TCS}}({\vec{\sigma }}_{\text{w}.\text{t}.})$ between a mutant sequence, ${\vec{\sigma }}_{\text{mutant}}$, and a wild-type sequence, ${\vec{\sigma }}_{\text{w}.\text{t}.}$. Once again, the sequence $\vec{\sigma }=(\sigma_{1},\sigma_{2},\ldots ,\sigma_{L})$ is a concatenated sequence of the HK EnvZ and the RR Spo0F, where only single-site mutations are made to the Spo0F in this present work. The coevolutionary landscape (Eq 1) as well as the HK/RR MSA sequences used to train the landscape are available in S1 File. Coevolutionary landscapes can also be used to identify TCS partner interactions within an organism [18, 41], i.e., which HKs and RRs preferentially interact. These approaches are consistent with earlier approaches that used information-based scores [16, 19, 42].

### Direct Information (DI)

Coevolution between residue pairs *i* and *j* can be quantified using the Direct Information (DI) [13, 20, 43, 44], a Kullback-Leibler divergence:

$$\begin{array}{l} DI_{ij}=\sum_{\sigma_{i}=1}^{q}\sum_{\sigma_{i}=1}^{q}P_{ij}^{\left(DCA\right)}(\sigma_{i},\sigma_{j})\text{log}\left(\frac{P_{ij}^{\left(DCA\right)}(\sigma_{i},\sigma_{j})}{P_{i}(\sigma_{i})P_{j}(\sigma_{j})}\right)\\ P_{ij}^{\left(DCA\right)}(\sigma_{i},\sigma_{j})=N\text{exp}(J_{ij}(\sigma_{i},\sigma_{j})+{\tilde{h}}_{i}(\sigma_{i})+{\tilde{h}}_{j}(\sigma_{j})) \end{array}$$ (2)

where $P_{ij}^{\left(DCA\right)}(\sigma_{i},\sigma_{j})$ is the inferred pair distribution between residues *i* and *j* with amino acids *σ*_*i*_ and *σ*_*j*_, respectively; *N* is the normalization; and ${\tilde{h}}_{i}(\sigma_{i})$ and ${\tilde{h}}_{j}(\sigma_{j})$ are chosen such that $P_{ij}^{\left(DCA\right)}(\sigma_{i},\sigma_{j})$ satisfies the marginalization conditions, $\sum_{\left\{\sigma_{j}\right\}}P_{ij}^{\left(DCA\right)}(\sigma_{i},\sigma_{j})=P_{i}(\sigma_{i})$ [13, 20, 43, 44]. The DI quantifies the informational entropy difference between the inferred pair distribution, $P_{ij}^{\left(DCA\right)}(\sigma_{i},\sigma_{j})$, with respect to a null model lacking pairwise correlations, *P*_*i*_(*σ*_*i*_)*P*_*j*_(*σ*_*j*_).

### Protein Purification

All protein-encoding genes were purchased from Genescript. EnvZ was inserted into a pET-32b cloning vector, including a TEV sequence, using the restriction sites Msc I and Nco I in the N-terminal and C-terminal, respectively. Spo0F was inserted into a pET-20b(+) cloning vector with restriction sites Nde I and Xho I in the N-terminal and C-terminal, respectively. See the S2 Text for more details on the DNA coding sequences that were used. The EnvZ and Spo0F plasmids were transformed into BL21(DE3)pLysS and C43 competent cells, respectively, and grown in LB media to an OD of 0.6. Protein expression was induced with the addition of 1mM IPTG for 4–5 hours at 37° C. Cells were harvested by centrifugation and resuspended in lysis buffer (50 mM Tris pH 8.0, 1M NaCl, 20 mM imidazole, 10% glycerol). EnvZ expressing cells were sonicated and lysate was separated via centrifugation at 20000 × *g*. EnvZ was purified with a (His)_6_-tag using Ni-NTA agarose (Qiagen) on a gravity flow column. EnvZ was bound to Ni-NTA agarose by placing the column on a rocking platform at 4°C for 1 hr. Bound EnvZ was subjected to 10 resin volumes of wash buffer (50 mM Tris pH 8.0, 1 M NaCl, 100 mM imidazole, 10% glycerol) and eluted in 2 resin volumes in buffer (50 mM Tris pH 8.0, 1M NaCl, 250 mM imidazole, 10% glycerol). Spo0F expressing cells were sonicated in buffer (50 mM Tris pH 8.0) and lysate was centrifuged at 20000 × *g*. The supernatant was filtered through a 20 kDa cut-off filter (Amicon Ultra-15 centrifugal filter). The flow-through was loaded onto a Q-column (GE Healthcare Life Sciences HiTrap Q HP) and the anion exchange chromatography was performed using a NaCl gradient up to 500mM NaCl. In each case, fractions containing protein were pooled together, concentrated through spin column centrifugation using a 3 kDa cutoff (Amicon Ultra-15 centrifugal filter). Concentrated protein was dialyzed (cutoff 3 kDa) against phosphorylation assay buffer (see buffer conditions below) overnight at 4°C. Protein purity was evaluated via SDS-PAGE.

### Phosphotransfer experiments

The phosphotransfer between EnvZ and Spo0F was measured using a radiolabeled ATP phosphotransfer assay. EnvZ and Spo0F were separately equilibrated in phosphorylation assay buffer (10 mM HEPES, 50 mM KCl, 10 mM MgCl_2_ and 0.1 mM EDTA). 100 mM ATP and 5 μCi [γ^32^P]ATP (6000 Ci/mmol) was added to the EnvZ sample to allow the autophosphorylation reaction to reach equilibrium. Equimolar amounts (2.5uM each final) of phosphorylated EnvZ and Spo0F were then combined to initiate the phosphotransfer reaction. The reactions were quenched with 4 × SDS-PAGE loading buffer (100mM Tris pH 6.8, 8% SDS, 0.2% bromophenol blue, 20% glycerol) using time points ranging from 1–90 minutes, loaded on a SDS poly-acrylamide gel, run at 100 V for 1.5 hours and set to dry for 16 hours. The dried gel was exposed to film for times ranging from 10–60 minutes depending on activity for visualization, and individual protein bands corresponding to phosphorylated Spo0F were quantitated on the ^32^P channel in liquid scintillant. The concentration of Spo0F-P (phosphorylated) was determined via the total counts per minutes (cpm) and the specific radioactive activity of each reaction mixture. Additionally, all mutant phosphotransfer assays were performed in parallel with wild-type Spo0F phosphotransfer to minimize any experimental variation in the EnvZ activity, and with mutants being run in duplicate. Reaction velocities for mutations were then calculated by fitting an exponential growth function to a complete progress curve and expressed as a ratio compared to the wild-type enzyme.

### Thermal stability through Differential Scanning Calorimetry (DSC) measurements

To verify if the introduced point mutations have an effect on the global protein stability, Differential Scanning Calorimetry (DSC) measurements were performed using a Microcal VP-Capilllary DSC Instrument, scanning from 20 to 100 °C. DSC measures the heat change associated with thermal unfolding at a constant rate, i.e., the thermal transition midpoint (T_m_) is obtained together with the change in enthalpy (Δ*H*_DSC_) upon unfolding of the protein. Data analyses were performed using the MicroCal Origin Software, and the change in the enthalpy of unfolding Δ*H*_DSC_ of mutated Spo0F proteins are plotted in Table 1. The data was collected at a 90 deg/hr scan rate using a protein concentration of 1mg/ml.

## Supporting information

S1 Text Selection of amino acids for Spo0F mutational sites. (DOCX)

S2 Text Full DNA coding sequences of EnvZ and Spo0F. (DOCX)

S1 Fig Point mutations selected to enhance phosphotransfer activity. The proxy for signal transfer efficiency (Eq 1 is plotted for all amino acid possibilities at each selected residue site on Spo0F (A-F), where Δ*H*_TCS_ < 0, Δ*H*_TCS_ > 0, and Δ*H*_TCS_ ≈ 0 denote mutations that are predicted to enhance, suppress, or have a neutral effect on phosphotransfer, respectively. A red circle is drawn around the amino acid identity of the wild-type protein, while a blue square is drawn around the amino acid identity of the point mutation.
(EPS)

S2 Fig Point mutations selected to suppress phosphotransfer activity. The proxy for signal transfer efficiency (Eq 1) is plotted for all amino acid possibilities at each selected residue site on Spo0F (A and B), where Δ*H*_TCS_ < 0, Δ*H*_TCS_ > 0, and Δ*H*_TCS_ ≈ 0 denote mutations that are predicted to enhance, suppress, or have a neutral effect on phosphotransfer, respectively. A red circle is drawn around the amino acid identity of the wild-type protein, while a blue square is drawn around the amino acid identity of the point mutation.
(EPS)

S3 Fig Point mutations selected for neutral effect on phosphotransfer activity. The proxy for signal transfer efficiency (Eq 1) is plotted for all amino acid possibilities at each selected residue site on Spo0F (A-D), where Δ*H*_TCS_ < 0, Δ*H*_TCS_ > 0, and Δ*H*_TCS_ ≈ 0 denote mutations that are predicted to enhance, suppress, or have a neutral effect on phosphotransfer, respectively. A red circle is drawn around the amino acid identity of the wild-type protein, while a blue square is drawn around the amino acid identity of the point mutation.
(EPS)

S4 Fig Comparison of Spo0F WT and mutant sequences with WT OmpR sequence. The Multiple Sequence Alignment (MSA) of Spo0F from *B*. *subtilis* and OmpR from *E*. *coli* are shown aligned with the secondary structure of the response regulator protein family. The locations of the point mutations examined in this study are highlighted in red. The asterisk labels the residues of Spo0F and OmpR that are identical (approximately 32% of the residues are identical). The “I” letter at the bottom denotes an identical mutation, i.e., the Spo0F mutation matches the amino acid found that that position in OmpR.
(EPS)

S1 File The compressed supporting information file contains 3 directories: (A) hmm_profiles, (B) database, and (C) code. (A) The hmm_profile directory contains the HMM profiles that are used to align Histidine Kinase DHp (PF00512) sequences and Response Regulator REC (PF00072) sequences to their respective MSAs. Note, our MSA of the HK proteins differs from the given PF00512 profile in that the first four residue sites of the MSA were removed. (B) The database directory contains HK/RR partner sequences used to train the Potts model. (C) The code directory contains the MATLAB code for calculating Eq 1. The user supplies sequence input in fasta format of the concatenated DHp/REC MSA for the HK/RR protein pair of interest. An example sequence (seq_test.txt) is provided. The code relies on several dependencies to be in the same directory (i.e., PottsModel.mat, Contacts.txt).
(ZIP)

## References

[1] Capra EJ, Perchuk BS, Lubin EA, Ashenberg O, Skerker JM, Laub MT. Systematic Dissection and Trajectory-Scanning Mutagenesis of the Molecular Interface That Ensures Specificity of Two-Component Signaling Pathways. PLoS Genetics. 2010;6(11):e1001220 10.1371/journal.pgen.1001220 21124821 PMC2991266

[2] Skerker JM, Perchuk BS, Siryaporn A, Lubin EA, Ashenberg O, Goulian M, et al Rewiring the Specificity of Two-Component Signal Transduction Systems. Cell. 2008;133(6):1043–54. 10.1016/j.cell.2008.04.040. 18555780 PMC2453690

[3] Podgornaia AI, Laub MT. Determinants of specificity in two-component signal transduction. Current Opinion in Microbiology. 2013;16(2):156–62. 10.1016/j.mib.2013.01.004 23352354

[4] Hoch JA. Two-component and phosphorelay signal transduction. Current Opinion in Microbiology. 2000;3(2):165–70. 10.1016/S1369-5274(00)00070-9. 10745001

[5] Stock AM, Robinson VL, Goudreau PN. Two-component signal transduction. Annual Review of Biochemistry. 2000;69(1):183–215. 10.1146/annurev.biochem.69.1.183 10966457

[6] Laub MT, Goulian M. Specificity in Two-Component Signal Transduction Pathways. Annual Review of Genetics. 2007;41:121–45. 10.1146/annurev.genet.41.042007.170548 18076326

[7] Casino P, Rubio V, Marina A. The mechanism of signal transduction by two-component systems. Current Opinion in Structural Biology. 2010;20(6):763–71. 10.1016/j.sbi.2010.09.010. 20951027

[8] Szurmant H, Hoch JA. Interaction fidelity in two-component signaling. Current Opinion in Microbiology. 2010;13(2):190–7. 10.1016/j.mib.2010.01.007. 20133181 PMC2847666

[9] Capra EJ, Laub MT. Evolution of two-component signal transduction systems. Annu Rev Microbiol. 2012;66:325–47. 10.1146/annurev-micro-092611-150039 . 22746333 PMC4097194

[10] Yamamoto K, Hirao K, Oshima T, Aiba H, Utsumi R, Ishihama A. Functional characterization in vitro of all two-component signal transduction systems from Escherichia coli. Journal of Biological Chemistry. 2005;280(2):1448–56. 10.1074/jbc.M410104200 15522865

[11] Laub MT, Biondi EG, Skerker JM. Phosphotransfer profiling: Systematic mapping of two-component signal transduction pathways and phosphorelays. Method Enzymol. 2007;423:531–48. 10.1016/S0076-6879(07)23026-5 17609150

[12] Schug A, Weigt M, Onuchic JN, Hwa T, Szurmant H. High-resolution protein complexes from integrating genomic information with molecular simulation. Proceedings of the National Academy of Sciences. 2009;106(52):22124–9. 10.1073/pnas.0912100106 20018738 PMC2799721

[13] Weigt M, White RA, Szurmant H, Hoch JA, Hwa T. Identification of direct residue contacts in protein-protein interaction by message passing. Proceedings of the National Academy of Sciences of the United States of America. 2009;106(1):67–72. 10.1073/pnas.0805923106 19116270 PMC2629192

[14] Li L, Shakhnovich EI, Mirny LA. Amino acids determining enzyme-substrate specificity in prokaryotic and eukaryotic protein kinases. Proceedings of the National Academy of Sciences. 2003;100(8):4463–8. 10.1073/pnas.0737647100 12679523 PMC153578

[15] White RA, Szurmant H, Hoch JA, Hwa T. Features of Protein–Protein Interactions in Two‐Component Signaling Deduced from Genomic Libraries In: Melvin I. Simon BRC, Alexandrine C, editors. Methods in Enzymology. Volume 422: Academic Press; 2007 p. 75–101. 10.1016/S0076-6879(06)22004-4 17628135

[16] Cheng RR, Morcos F, Levine H, Onuchic JN. Toward rationally redesigning bacterial two-component signaling systems using coevolutionary information. Proceedings of the National Academy of Sciences of the United States of America. 2014;111(5):E563–E71. 10.1073/pnas.1323734111 24449878 PMC3918776

[17] Burger L, van Nimwegen E. Accurate prediction of protein-protein interactions from sequence alignments using a Bayesian method. Mol Syst Biol. 2008;4(165). 10.1038/msb4100203 18277381 PMC2267735

[18] Cheng RR, Nordesjo O, Hayes RL, Levine H, Flores SC, Onuchic JN, et al Connecting the Sequence-Space of Bacterial Signaling Proteins to Phenotypes Using Coevolutionary Landscapes. Mol Biol Evol. 2016;33(12):3054–64. 10.1093/molbev/msw188 . 27604223 PMC5100047

[19] Procaccini A, Lunt B, Szurmant H, Hwa T, Weigt M. Dissecting the Specificity of Protein-Protein Interaction in Bacterial Two-Component Signaling: Orphans and Crosstalks. PloS one. 2011;6(5):e19729 10.1371/journal.pone.0019729 21573011 PMC3090404

[20] Morcos F, Pagnani A, Lunt B, Bertolino A, Marks DS, Sander C, et al Direct-coupling analysis of residue coevolution captures native contacts across many protein families. Proceedings of the National Academy of Sciences. 2011;108(49):E1293–E301. 10.1073/pnas.1111471108 22106262 PMC3241805

[21] Ekeberg M, Lovkvist C, Lan YH, Weigt M, Aurell E. Improved contact prediction in proteins: Using pseudolikelihoods to infer Potts models. Phys Rev E. 2013;87(1):012707 10.1103/PhysRevE.87.012707 23410359

[22] Ovchinnikov S, Park H, Varghese N, Huang PS, Pavlopoulos GA, Kim DE, et al Protein structure determination using metagenome sequence data. Science. 2017;355(6322):294–7. 10.1126/science.aah4043 28104891 PMC5493203

[23] Marks DS, Hopf TA, Sander C. Protein structure prediction from sequence variation. Nature biotechnology. 2012;30(11):1072–80. Epub 2012/11/10. 10.1038/nbt.2419 . 23138306 PMC4319528

[24] de Juan D, Pazos F, Valencia A. Emerging methods in protein co-evolution. Nature reviews Genetics. 2013;14(4):249–61. Epub 2013/03/06. 10.1038/nrg3414 . 23458856

[25] Burbulys D, Trach KA, Hoch JA. Initiation of sporulation in B. subtilis is controlled by a multicomponent phosphorelay. Cell. 1991;64(3):545–52. 10.1016/0092-8674(91)90238-T. 1846779

[26] Casino P, Rubio V, Marina A. Structural Insight into Partner Specificity and Phosphoryl Transfer in Two-Component Signal Transduction. Cell. 2009;139(2):325–36. 10.1016/j.cell.2009.08.032. 19800110

[27] Figliuzzi M, Jacquier H, Schug A, Tenaillon O, Weigt M. Coevolutionary Landscape Inference and the Context-Dependence of Mutations in Beta-Lactamase TEM-1. Molecular Biology and Evolution. 2016;33(1):268–80. 10.1093/molbev/msv211 26446903 PMC4693977

[28] Starr TN, Thornton JW. Epistasis in protein evolution. Protein Sci. 2016;25(7):1204–18. 10.1002/pro.2897 26833806 PMC4918427

[29] Hopf TA, Ingraham JB, Poelwijk FJ, Scharfe CPI, Springer M, Sander C, et al Mutation effects predicted from sequence co-variation. Nature biotechnology. 2017;35(2):128–35. 10.1038/nbt.3769 28092658 PMC5383098

[30] Gueudre T, Baldassi C, Zamparo M, Weigt M, Pagnani A. Simultaneous identification of specifically interacting paralogs and interprotein contacts by direct coupling analysis. Proceedings of the National Academy of Sciences of the United States of America. 2016;113(43):12186–91. 10.1073/pnas.1607570113 27729520 PMC5087065

[31] Podgornaia AI, Laub MT. Pervasive degeneracy and epistasis in a protein-protein interface. Science. 2015;347(6222):673–7. 10.1126/science.1257360 25657251

[32] Tabor JJ, Levskaya A, Voigt CA. Multichromatic control of gene expression in Escherichia coli. J Mol Biol. 2011;405(2):315–24. 10.1016/j.jmb.2010.10.038 . 21035461 PMC3053042

[33] Whitaker WR, Davis SA, Arkin AP, Dueber JE. Engineering robust control of two-component system phosphotransfer using modular scaffolds. Proceedings of the National Academy of Sciences of the United States of America. 2012;109(44):18090–5. 10.1073/pnas.1209230109 23071327 PMC3497815

[34] Ganesh I, Ravikumar S, Lee SH, Park SJ, Hong SH. Engineered fumarate sensing Escherichia coli based on novel chimeric two-component system. J Biotechnol. 2013;168(4):560–6. 10.1016/j.jbiotec.2013.09.003 . 24056083

[35] Schmid SR, Sheth RU, Wu A, Tabor JJ. Refactoring and Optimization of Light-Switchable Escherichia coli Two-Component Systems. Acs Synth Biol. 2014;3(11):820–31. 10.1021/sb500273n 25250630

[36] Hansen J, Benenson Y. Synthetic biology of cell signaling. Nat Comput. 2016;15(1):5–13. 10.1007/s11047-015-9526-1

[37] Mukhopadhyay D, Sen U, Zapf J, Varughese KI. Metals in the sporulation phosphorelay: manganese binding by the response regulator Spo0F. Acta Crystallographica Section D. 2004;60(4):638–45. 10.1107/S0907444904002148 15039551

[38] Zhang Y. I-TASSER server for protein 3D structure prediction. BMC Bioinformatics. 2008;9(1):40 10.1186/1471-2105-9-40 18215316 PMC2245901

[39] Finn RD, Bateman A, Clements J, Coggill P, Eberhardt RY, Eddy SR, et al Pfam: the protein families database. Nucleic Acids Research. 2014;42(D1):D222–D30. 10.1093/nar/gkt1223 24288371 PMC3965110

[40] Skerker JM, Prasol MS, Perchuk BS, Biondi EG, Laub MT. Two-component signal transduction pathways regulating growth and cell cycle progression in a bacterium: A system-level analysis. Plos Biol. 2005;3(10):1770–88. 10.1371/journal.pbio.0030334 16176121 PMC1233412

[41] Bitbol AF, Dwyer RS, Colwell LJ, Wingreen NS. Inferring interaction partners from protein sequences. Proceedings of the National Academy of Sciences of the United States of America. 2016;113(43):12180–5. 10.1073/pnas.1606762113 . 27663738 PMC5087060

[42] Boyd JS, Cheng RR, Paddock ML, Sancar C, Morcos F, Golden SS. A Combined Computational and Genetic Approach Uncovers Network Interactions of the Cyanobacterial Circadian Clock. Journal of Bacteriology. 2016;198(18):2439–47. 10.1128/JB.00235-16 27381914 PMC4999937

[43] Morcos F, Jana B, Hwa T, Onuchic JN. Coevolutionary signals across protein lineages help capture multiple protein conformations. Proceedings of the National Academy of Sciences. 2013;110(51):20533–8. 10.1073/pnas.1315625110 24297889 PMC3870752

[44] dos Santos RN, Morcos F, Jana B, Andricopulo AD, Onuchic JN. Dimeric interactions and complex formation using direct coevolutionary couplings. Sci Rep-Uk. 2015;5:13652 10.1038/srep13652 26338201 PMC4559900

[45] Schrodinger L. The PyMOL Molecular Graphics System, Version 1.8 2015.

