# Supplementary material for: Designing bacterial signaling interactions with coevolutionary landscapes
Source: PLoS One. 2018 Aug 20;13(8):e0201734. doi: 10.1371/journal.pone.0201734 (PMC6101370; doi:10.1371/journal.pone.0201734)
Supplement: S1 Text — (DOCX) [file pone.0201734.s001.docx]

**Selection of amino acids for Spo0F mutational sites**

As discussed in the main text, the Spo0F mutational sites were selected from the subset of residue sites that form the TCS binding interface and highly coevolve with residues of the HK over the course of natural selection.

The proxy for signal transfer efficiency was used to find amino acids to enhance, suppress, or have a neutral effect on the activity between EnvZ and Spo0F, i.e., amino acids that satisfy the conditions $\Delta H_{\text{TCS}}<0$, $\Delta H_{\text{TCS}}>0$, and $\Delta H_{\text{TCS}}\approx0$, respectively. For each mutational site, there were typically several amino acid point mutations that satisfied the desired mutational effect (S1-S3 Figs).

To narrow the selection of mutations for cases in which $\Delta H_{\text{TCS}}<0$, several of the mutations were also chosen to match the amino acid identity of OmpR at the same residue positions. The receiver domain of Spo0F shares a 32% sequence identity with the receiver domain of OmpR, the cognate partner of EnvZ. Of the point mutations selected to enhance phosphotransfer, 5 of the 6 matched the amino acids of OmpR at the respective positions. The lone exception was the I108L mutation, which led to the greatest enhancement in the measured phosphotransfer. It should be noted that simply selecting residues to enhance the activity by copying the corresponding residue of a known partner to the kinase is insufficient. For example, K56M and I57L match amino acid identity with OmpR and are found to have comparable and diminished activity, respectively. A full comparison between the multiple sequence alignment of Spo0F and OmpR highlights the similarities and differences between the selected point mutations and the cognate partner OmpR (S4 Fig).
