# Supplementary material for: Designing bacterial signaling interactions with coevolutionary landscapes
Source: PLoS One. 2018 Aug 20;13(8):e0201734. doi: 10.1371/journal.pone.0201734 (PMC6101370; doi:10.1371/journal.pone.0201734)
Supplement: S2 Text — (DOCX) [file pone.0201734.s002.docx]

**Full DNA coding sequences of EnvZ and Spo0F**

EnvZ was inserted into a pET-32b cloning vector, including a TEV sequence, using restriction sites Msc I and Nco I in the N-terminal and C-terminal, respectively. The DNA sequence for EnvZ used in this study:

ATGCATCATCATCATCATCATAGCAGCGGCGAAAATCTGTACTTCCAGGGTGCGTTCAACCACATGGCGGCAGGCGTCAAACAACTGGCGGATGACCGTACCCTGCTGATGGCCGGTGTGAGCCATGATCTGCGCACCCCGCTGACGCGTATCCGCCTGGCGACGGAAATGATGAGCGAACAGGATGGCTATCTGGCCGAATCTATTAACAAAGACATCGAAGAATGCAATGCAATTATCGAACAGTTTATCGATTACCTGCGTACCGGTCAAGAAATGCCGATGGAAATGGCAGACCTGAACGCTGTCCTGGGTGAAGTGATTGCGGCCGAATCCGGCTATGAACGCGAAATTGAAACGGCGCTGTATCCGGGCAGCATCGAAGTGAAAATGCACCCGCTGTCAATTAAACGTGCGGTTGCCAACATGGTGGTTAATGCAGCTCGCTATGGCAACGGTTGGATCAAAGTCAGCTCTGGCACCGAACCGAATCGTGCTTGGTTCCAGGTTGAAGATGACGGTCCGGGCATCGCACCGGAACAGCGTAAACACCTGTTTCAACCGTTCGTTCGTGGCGATAGTGCACGCACCATTTCCGGCACGGGTCTGGGCCTGGCTATTGTTCAACGTATCGTGGATAACCACAATGGTATGCTGGAACTGGGCACCTCAGAACGTGGCGGTCTGTCGATTCGCGCGTGGCTGCCGTAA

coding for the amino acid sequence:

*MHHHHHHSSGENLYFQGAFNHMAAGVKQLADDRTLLMAGVSHDLRTPLTRIRLATEMMSEQDGYLAESINKDIEECNAIIEQFIDYLRTGQEMPMEMADLNAVLGEVIAAESGYEREIETALYPGSIEVKMHPLSIKRAVANMVVNAARYGNGWIKVSSGTEPNRAWFQVEDDGPGIAPEQRKHLFQPFVRGDSARTISGTGLGLAIVQRIVDNHNGMLELGTSERGGLSIRAWLP*

Spo0F was inserted into a pET-20b(+) cloning vector with restriction sites Nde I and Xho I in the N-terminal and C-terminal, respectively. The DNA sequence for Spo0F used in current work is:

ATGAATGAAAAAATCCTGATTGTGGACGACCAGTATGGCATCCGTATCCTGCTGAATGAAGTGTTTAATAAAGAAGGCTACCAGACCTTTCAGGCGGCCAACGGCCTGCAAGCGCTGGATATTGTCACGAAAGAACGTCCGGATCTGGTGCTGCTGGACATGAAAATTCCGGGCATGGATGGTATTGAAATCCTGAAACGTATGAAAGTGATTGACGAAAATATCCGCGTTATTATCATGACCGCGTATGGCGAACTGGATATGATTCAGGAAAGCAAAGAACTGGGTGCACTGACGCATTTTGCTAAACCGTTCGATATTGACGAAATCCGCGACGCCGTTAAAAAATACCTGCCGCTGAAATCTAACTAA

coding for the amino acid sequence:

MMNEKILIVDDQYGIRILLNEVFNKEGYQTFQAANGLQALDIVTKERPDLVLLDMKIPGMDGIEILKRMKVIDENIRVIIMTAYGELDMIQESKELGALTHFAKPFDIDEIRDAVKKYLPLKSN
