# Supplementary material for: Designing bacterial signaling interactions with coevolutionary landscapes
Source: PLoS One. 2018 Aug 20;13(8):e0201734. doi: 10.1371/journal.pone.0201734 (PMC6101370; doi:10.1371/journal.pone.0201734)
Supplement: S1 File — (A) The hmm_profile directory contains the HMM profiles that are used to align Histidine Kinase DHp (PF00512) sequences and Response Regulator REC (PF00072) sequences to their respective MSAs. Note, our MSA of the HK proteins differs from the given PF00512 profile in that the first four residue sites of the MSA were removed. (B) The database directory contains HK/RR partner sequences used to train the Potts model. (C) The code directory contains the MATLAB code for calculating Eq 1. The user supplies sequence input in fasta format of the concatenated DHp/REC MSA for the HK/RR protein pair of interest. An example sequence (seq_test.txt) is provided. The code relies on several dependencies to be in the same directory (i.e., PottsModel.mat, Contacts.txt). (ZIP) [file pone.0201734.s007.zip › S1 File/README.docx]

The compressed file contains 3 directories: (1) hmm_profiles, (2) database, and (3) code

(1) Contains the HMM profiles that are used to align Histidine Kinase DHp (PF00512) sequences and Response Regulator REC (PF00072) sequences to their respective MSA.
Note: The first 4 positions of the HK MSA are removed.

(2) The database of the concatenated HK/RR partner sequences. Sequences were paired based on the operon assumption. This database was used to construct the Potts model for HK/RR coevolution.

(3) This directory contains the MATLAB code for calculating Equation 1. The user supplies sequence input in fasta format of the concatenated DHp/REC MSA for the HK/RR protein pair of interest. See Materials and Methods and the example input sequence (seq_test.txt).

The code relies on several dependencies to be in the same directory (i.e., PottsModel.mat, Contacts.txt).

For assistance, please contact Ryan R. Cheng ([ryan.r.cheng@gmail.com](mailto:ryan.r.cheng@gmail.com)) or Faruck Morcos ([faruckm@utdallas.edu](mailto:faruckm@utdallas.edu)).
